# Atrazine Affects Phosphoprotein and Protein Expression in MCF-10A Human Breast Epithelial Cells

**DOI:** 10.3390/ijms151017806

**Published:** 2014-10-01

**Authors:** Peixin Huang, John Yang, Qisheng Song, David Sheehan

**Affiliations:** 1 Department of Agriculture & Environmental Science, Lincoln University of Missouri, Jefferson City, MO 65120, USA; E-Mail: phuang@cmh.edu; 2 Division of Plant Sciences, University of Missouri, Columbia, MO 65211, USA

**Keywords:** Atrazine, MCF-10A cells, 2DE, protein expression, ANP32A

## Abstract

Atrazine, a member of the 2-chloro-s-triazine family of herbicides, is the most widely used pesticide in the world and often detected in agriculture watersheds. Although it was generally considered as an endocrine disruptor, posing a potential threat to human health, the molecular mechanisms of atrazine effects remain unclear. Using two-dimensional gel electrophoresis, we identified a panel of differentially expressed phosphoproteins and total proteins in human breast epithelial MCF-10A cells after being exposed to environmentally relevant concentrations of atrazine. Atrazine treatments for 6 h resulted in differential expression of 4 phosphoproteins and 8 total-proteins as compared to the control cells (>1.5-fold, *p <* 0.05). MALDI-TOF MS/MS analysis revealed that the differentially expressed proteins belong to various cellular compartments (nucleus, cytosol, membrane) and varied in function, including those regulating the stress response such as peroxiredoxin I, HSP70 and HSP27; structural proteins such as tropomyosin and profilin 1; and oncogenesis proteins such as ANP32A. Six of the 12 identified proteins were verified by quantitative PCR for their transcript levels. The most up-regulated phosphoprotein by atrazine treatment, ANP32A, was further analyzed for its expression, distribution and cellular localization using Western blot and immunocytochemical approaches. The results revealed that ANP32 expression after atrazine treatment increased dose and time dependently and was primarily located in the nucleus. This study may provide new evidence on the potential toxicity of atrazine in human cells.

## 1. Introduction

Atrazine, a member of the 2-chloro-s-triazine family of herbicides, is the most widely used pesticide in the world [1,2]. It is the most common pesticide present in ground and surface water [3,4,5,6,7,8,9] and chemically persistent in the environment [10]. Atrazine at a concentration of 20 μg/L (20 ppb) is commonly detected in surface water and the concentration can be as high as 700 μg/L (700 ppb) [11,12]. Although the Environmental Protection Agency (EPA) monitors and enforces a maximum contaminant level (MCL) of 3 μg/L (3.0 ppb) of atrazine in public drinking water (Safe Drinking Water Act, 1991), a recent study by United States Department of Agriculture (USDA) reported that the annual mean atrazine concentration has exceeded the MCL in most drinking source water and the level in ground water could reach up to 6.5 μg/L (6.5 ppb) [13].

Elevated atrazine in the environment is of health concern. Atrazine was reported to be an endocrine disrupting compound (EDC), having adverse impacts on the central nervous system [14,15,16], the endocrine system [17,18,19,20,21] and the immune system [22,23]. Even though atrazine has been shown to affect the reproductive health of rats [18], pigs [24], fish [21] and amphibians [19,20,25], its effects on mammals, particularly on humans are little studied and largely unknown.

Epidemiologic studies have linked long-term exposure of triazine herbicides to increased risk of ovarian cancer in female farm workers in Italy [26] and breast cancer in Kentucky [27]. In addition, atrazine was also reported to lead to tumor development in the mammary gland and reproductive organs of female F344 rats [28] and to cause an earlier onset of mammary and pituitary tumors [29] in Sprague-Dawley rats, a typical response to exogenously administered estrogens [30]. In overall evaluation of animal carcinogenicity, the International Agency for Research on Cancer (IARC) [31] concluded that the Sprague-Dawley rat mammary gland tumors following exposure to atrazine involved a non-DNA reactive, hormonally mediated mechanism. A similar conclusion was made by the U.S. EPA [32].

Although a few studies on the effects of atrazine in animals and humans were reported, the studies at molecular level, especially at protein level are seldom. Recently, proteomic analysis is regarded as a powerful tool in investigating a large-scale of gene expression at protein level, and many proteomic studies on the toxicity of xenobiotics, especially on chemical carcinogens were reported [33]. High throughput proteome profiling and bioinformatics technology would allow examining the changes in protein expression of human cells in response to xenobiotics, and therefore, identification of the differentially expressed proteins could lead to a better understanding of the molecular mechanisms involved in toxicity of xenobiotics. Lasserre *et al.* [34] suggested that atrazine could affect protein expression, including those proteins involved in regulating oxidative stress and structural proteins, in MCF-7 breast cancer cells using 2DE in combined with MALDI-TOF-TOF analysis.

In this study, human breast epithelial MCF-10A cells were used as a model because atrazine has been reported to increase the incidence of mammary tumors in female Sprague-Dawley rats [29] and human breast cancers [27]. Moreover, MCF-10A cells are not tumorigenic and considered to be “normal” breast epithelial cells, thus it provides an excellent system for studying the tumorigenic effect of atrazine on “normal” breast cells. This research was to identify and verify the differentially expressed proteins induced by atrazine at environmental relevant concentrations in MCF-10A cells using proteomic, molecular and immunological methods. The data presented in this study would provide insights of the toxicological mechanisms of atrazine on human cells.

## 2. Results and Discussion

### 2.1. 2-DE Analysis

**Figure 1:**
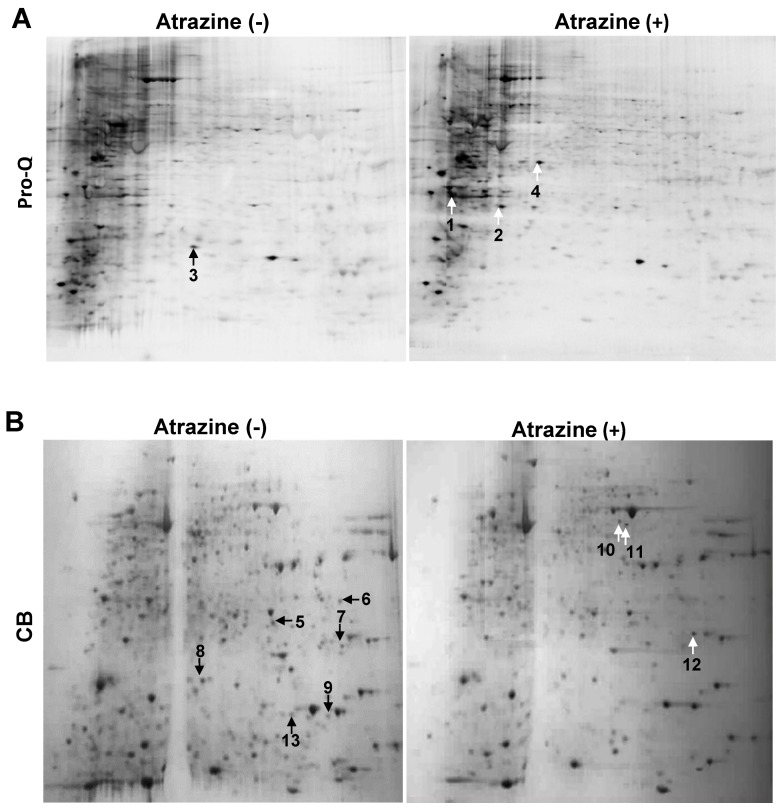
2D gel identification of differentially expressed phosphoproteins and total proteins in MCF-10A cells treated with or without atrazine. MCF-10A cells were treated with or without 0.1 µg/mL atrazine for 6 h, harvested and lysed. After centrifugation, proteins in the resulting supernatants were analyzed by 2D gel electrophoresis and stained with Pro-Q Diamond Phosphoprotein Gel Stain (**A**), followed by CB staining (**B**). Acquired images were repetitive. The data shown are representative of three separate experiments. Differentially expressed protein spots were labeled with arrows (black: under-expressed spots; white: over-expressed spots) and numbers, which are the same as those in Table 1.

To investigate the early events of atrazine-treated MCF-10A cells, the cells were treated for 3, 6 and 12 h with 0.1 µg/mL (100 ppb) atrazine, an environmental relevant concentration [34]. At the end of the incubation, proteins were extracted from the cells and subjected to 2D gel electrophoresis, followed by Pro-Q staining, which detects the fluorescence of phosphoserine-, phosphothreonine-, and phosphotyrosine-containing proteins directly in SDS-polyacryamide gels [35]. ProQ stained 2D gel maps revealed 4 differentially displayed phosphoprotein spots, with 3 increased (white arrows, labeled as #1, 2, 4) and 1 decreased (black arrow, labeled as #3) (Figure 1A). To further examine the atrazine effects on differential expressed total protein, the Pro-Q stained 2D gels were subjected to Coomassie Brilliant Blue (CB) G-250 staining. CB staining showed 9 differentially expressed proteins (Figure 1B) (*p <* 0.05, 2-sided), of which, 3 were increased (white arrows) and 6 decreased (black arrows) by the treatment. The gel images from the samples after 6 h atrazine treatment were shown since they represented the early events of differential phosphoprotein and total protein expression under the current experimental conditions (Figure 1).

### 2.2. Protein Identification

The differentially expressed phosphoproteins were localized by overlapping the Pro-Q stained images with the images of CB stained gels. The localized phosphoprotein and total protein spots were punched out, trypsinated, and subjected to MALDI-TOF/TOF MS and MS/MS analysis. The MS fingerprints were then used to identify proteins in the NCBInr protein databank. In this study, we focused on the most over- or under-expressed 13 spots as shown in Table 1. All 13 protein spots were identified by at least two peptides with a sufficiently high Mascot score and ion score, and had positive matches to 12 different proteins in the databank with Mascot scores ranging from 124 to 646 (Mascot score > 66 represents significance at *p* < 0.05). Among the 12 differentially expressed proteins, a heat shock protein 70 kDa was identified in two protein spots, suggesting the presence of isoforms and/or post-translational modifications that affect the pI and/or the molecular mass of the protein (Table 1 and Figure 1). The numbers of the phospoprotein and total protein spots in Table 1 were labeled in the same way as those in Figure 1.

**Table 1:** Differentially expressed proteins identified in MCF-10A cells by 2DE.

Spot #	Putative Protein Name	Name of Gene	Score	MW (kDa)	PI	Ion Score	Peptides
1	Acidic leucine-rich nuclear phosphoprotein 32 family, member A (gi: 5453880)	*ANP32A*	129	29	3.99	29	K.LLPQLTYLDGYDR.E
						85	K.SLDLFNCEVTNLNDYR.E
2	Heat shock protein beta-1 (gi: 4504517)	*HSPβ1*	160	23	5.98	20	R.GPSWDPER.D
						55	R.LFDQAFGLPR.L
3	Nucloside Diphosphate Kinase A isoform b (gi: 4557797)	*NME1*	602	35	5.83	63	R.GDFCIQVGR.N
						59	K.DRPFFAGLVK.Y
						83	R.TFIAIKPDGVQR.G
						94	R.VMLGETNPADSKPGTIR.G
4	Pyruvate kinase, various isoforms (gi: 35505)	*PDK*	425	58	7.58	63	R.LDIDSPPITAR.N
						50	R.LNFSHGTHEYHAETIK.N
						77	K.IYVDDGLISLQVK.Q
						46	R.RFDEILEASDGIMVAR.G
5	Tropomyosin alpha 3 chain isoform 2 (gi: 24119203)	*TPM3*	543	32	4.75	39	K.LVIIEGDLER.T
						80	R.IQLVEEELDR.A
						80	R.KLVIIEGDLER.T
						29	R.EQAEAEVASLNR.R
6	Guanine nucleotide-binding protein subunit beta-2-like 1 (gi: 5174447)	*GNB2L1*	646	35	7.6	67	R.VWQVTIGTR.R
						77	R.DETNYGIPQR.R
						133	K.YTVQDESHSEWVSCVR.F
						82	K.HLYTLDGGDIINALCFSPNR.Y
7	Transgelin-2 (gi: 4507357)	*TAGLN2*	232	23	8.4	42	K.NVIGLQMGTNR.G
						76	R.TLMNLGGLAVAR.D
						40	K.QMEQISQFLQAAER.Y
8	Stathmin 1 (gi: 5031851)	*STMN1*	166	17	5.76	102	R.ASGQAFELILSPR.S
						30	R.SKESVPEFPLSPPK.K
9	Profilin-1 (gi: 4826898)	*PFN1*	177	15	8.4	41	K.CYEMASHLR.R
						82	R.DSLLQDGEFSMDLR.T
10	Heat shock protein 70 kDa protein 4 (gi: 62087882)	*HSPA4*	124	71	5.44	80	K.DAGTIAGLNVLR.I
11			219			59	R.FEELNADLFR.G
						78	K.STAGDTHLGGEDFDNR.M
						103	K.TVTNAVVTVPAYFNDSQR.Q
12	Peroxiredoxin-1 (gi: 4505591)	*PRDX1*	276	22	8.3	33	K.ADEGISFR.G
						57	K.IGHPAPNFK.A
						81	R.QITVNDLPVGR.S
						28	K.HGEVCPAGWKPGSDTIKPDVQK.S
13	60S acidic ribosomal P12 (gi: 4506671)	*RPS12*	397	12	4.42	86	K.ILDSVGIEADDDRLNK.V
						103	R.YVASYLLAALGGNSSPSAK.D
						123	K.LASVPAGGAVAVSAAPGSAAPAAGSAPAAAEEK.K

Spot #: Protein spots were numbered exactly as those presented in Figure 1. Score: Mascot MS protein score obtained by analysis based on PMF (peptide mass fingerprint). Score greater than 66 indicates identity or extensive homology. MW: Predicted molecular weight (MW) in dalton (Da) according to protein sequence. PI: Predicted isoelectric point (PI) of the protein according to sequence. Ion Score: Mascot MS/MS ion score obtained by analysis based on MS/MS spectrum. Ion scores greater than 26 and 43 indicate homology and identity respectively.

To our knowledge, this is first report that investigated the effects of atrazine, an endocrine disrupting compound, on the protein and phosphoprotein expression levels in MCF-10A non-tumorigenic human breast epithelial cells. Atrazine has been reported to pose a variety of toxicological effects, including immunotoxicity [22,23], neurotoxicity [15,16], developmental toxicity [17,36], reproductive toxicity [18,19,20] and carcinogenesis [37]. This study revealed 12 differentially expressed phospho- and total proteins in response to atrazine treatment, which are involved in signaling, protein translation, gene transcription, structural stability, stress regulation, and glycolytic metabolism. In agreement with gene regulation of human cells affected by atrazine in a large scale reported by Dooley *et al.* [38], our results indicate that the differentially expressed proteins were not located in a single compartment of the cells (Table 2) and therefore the cell functions were differentially affected, indicating that atrazine targeted neither a unique compartment nor a single process in MCF-10A cells.

**Table 2:** Densitometry analysis of differentially expressed proteins in the atrazine-treated and control MCF-10A cells. The up- or down-regulated proteins were expressed as % of control.

Gene name	Cellular localization	Category	Function	Expression (% control)	*p* value
*NME1*	Cytoplasm; Nucleus	Metabolism	Major role in the synthesis of nucleoside triphosphates other than ATP	32	0.031
*PDK*	Mitochondrion		Plays a key role in glycolysis	342	0.024
*STMN1*	Cytoplasm; Cytoskeleton	Signalization	Involved in the regulation of microtubule (MT)	27	0.047
*GNB2L1*	Cell/Peripheral membrane; Cytoplasm		Involved in the recruitment, assembly and/or regulation of a variety of signaling molecules	23	0.035
*RPS12*	Ribosome	Translation	Plays an important role in the elongation step of protein synthesis	30	0.042
*ANP32A*	Nucleus; Cytoplasm; ER	Transcription	Implicated in some cellular processes, including proliferation, differentiation, and apoptosis	470	0.027
*PFN1*	Cytoplasm; Cytoskeleton	Structure	Binds to actin and affects the structure of the cytoskeleton	21	0.033
*TAGLN2*	Nuclear/plasma membrane		Binds to actin, calmodulin, troponin C and tropomyosin	19	0.014
*TPM3*	Cytoplasm; Cytoskeleton		Binds to actin filaments in muscle and non-muscle cells	33	0.039
*PRDX1*	Cytoplasm	Catabolism	Involved in redox regulation of the cell	260	0.040
*HSPβ1*	Cytoplasm	Chaperone	ATP-independent chaperone in protein folding	327	0.012
*HSPA4*	Cytoplasm		HSP 70 kDa is a chaperone	350	0.022

Reversible protein phosphorylation is known to play an important role in regulating various cell processes, such as cell growth, differentiation, migration, metabolism, and apoptosis. Analysis of Pro-Q Diamond stained 2D gels (Figure 1A) showed that 3 out of 4 identified phosphoproteins were over-expressed as a result of atrazine treatment, including ANP32A, HSPβ1 and PDK. Increased phosphorylation of these proteins suggested that atrazine may trigger some signal transduction pathways in MCF-10A cells. In contrast to these phosphoproteins, 6 out of 9 modulated total proteins were under-expressed (Figure 1B), which was in agreement with findings by Lasserre *et al.* [34], who reported that atrazine treatment seemed to decrease the activity of MCF-7 breast cancer cells with an 88% decrease in total modulated proteins.

### 2.3. Classification of Identified Proteins 

To obtain an overview of the atrazine effect on the proteome of MCF-10A human cells, the identified proteins were classified with respect to their subcellular localization and biological processes (Table 2) based on information from UniprotKB. All identified proteins were intracellular proteins and mostly distributed in the cytoplasm, nucleus, mitochondrial, ribosome, and membrane. The identified proteins were involved in various processes, primarily in cell structure [profilin-1 (PFN1), transgelin-2 (TAGLN2), and tropomyosin (TPM3)], stress response [heat shock protein 70 kDa protein 4 (HSPA4), heat shock protein beta-1 (HSPβ1) and peroxiredoxin-1 (PRDX1)], and energy metabolism [(nucleoside diphosphate kinase A isoform b (NME1) and pyruvate kinase (PDK)] (Table 2). The remaining proteins are involved in signaling [stathmin 1 (STMN1) and guanine nucleotide-binding protein subunit beta-2-like 1 (GNB2L1)], protein translation [60S acidic ribosomal P12 (RPS12)] and gene transcription [acidic leucine-rich nuclear phosphoprotein 32 family, member A (ANP32A)], are most likely to be the early event of MCF-10A cell response to atrazine treatment.

The cytoskeletal network plays an important role in cell shape and locomotion, which in turn are thought to be involved in growth control, such as division and proliferation. Changes in the intermediate filaments and actin induced by atrazine may affect cell morphology [33]. In this study, the changes in the expression of proteins to participate in cell proliferation (ANP32A, spot #1), tubulin stability (STMN1, spot #8), actin binding for skeleton structure stability (TAGLN2; spot #7; PFN1, spot #9), and actin filament stabilization (TMP3, spot #5) were observed. Among the 9 differentially expressed total proteins (Figure 1B), three involved in cell structure stability were inhibited by atrazine (Table 3). Inhibition of the structural proteins may result from the reduced protein synthesis, such as RPS12 (spot #13) that plays an essential role in the elongation step of protein synthesis.

**Table 3:** Primers used in qPCR for verifying the identified proteins candidates.

Primer Name	Forward	Reverse
Transgelin-2	5'-TGGCTTTGGGCAGCTTGGCA-3'	5'-TCCCTCCTGCAGCTGGCTCT-3'
Profilin-1	5'-CAAGAGCACCGGTGGGGCC-3'	5'-GAACGCCGAAGGTGGGAGGC-3'
NDP kinase A	5'-ACCGTCCATTCTTTGCCGGCC-3'	5'-CGAGCATGACTCGGCCCGTC-3'
Pyruvate kinase	5'-GCAGCGCCACTAAGCCGTGA-3'	5'-AGCGGCCAGTTGTGGTCAGC-3'
ANP32A	5'-TGCGGAACAGGACGCCCTCT-3'	5'-TGCGATTGAGGTGAGGCCTACGT-3'
HSPβ1	5'-GTCGCAGTGGTTAGGCGGCA-3'	5'-GACATCCAGGGACACGCGCC-3'
NAPDH (CK)	5'-GCCTTCCGTGTCCCCACTGC-3'	5'-CCTCCGACGCCTGCTTCACC-3'

### 2.4. Atrazine Effects on the Phosphoproteome and Proteome of MCF-10A Human Cells

Densitometric analysis of relative expression of identified proteins (Table 2) showed that atrazine treatment enhanced the phosphorylation of 3 proteins, including ANP32A, HSPβ1 and PDK, and inhibited the phosphorylation of NME1 (Figure 1A and Table 2), but had no effect on their total protein levels. The CB stained 2D gels revealed 9 differentially expressed proteins, but only 3 were elevated by atrazine treatment (Figure 1B and Table 2).

### 2.5. qPCR Verification of Transcriptional Regulation of the Identified Proteins

**Figure 2:**
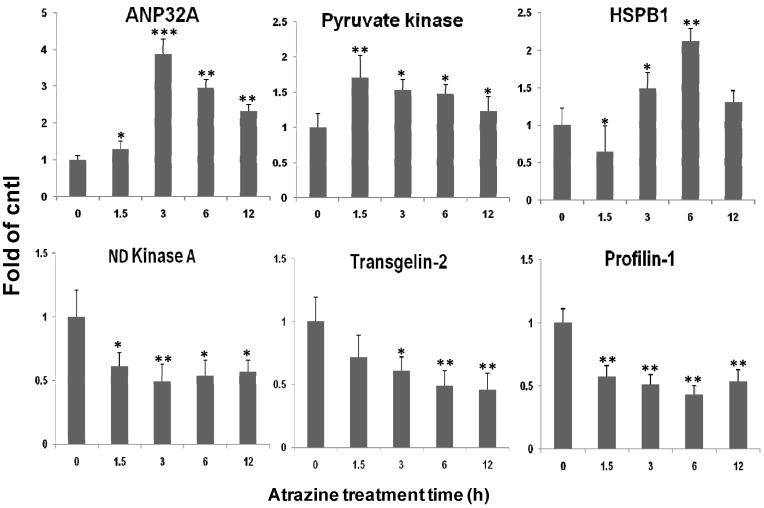
Real-time PCR analysis of transcript levels of 6 among 12 identified proteins. The MCF-10A cells were incubated in the presence or absence of 0.1 µg/mL atrazine for the indicated time periods. Total RNA was isolated for qPCR analysis of transcription levels for all 6 identified proteins. The GAPDH gene was used for normalization of the compared templates. The data represent the mean ± SD of three biological samples. * *p <* 0.05, ** *p <* 0.01, *** *p <* 0.001 *versus* control.

To confirm that the quantitative changes in protein levels registered on 2D gels (Figure 1) in response to atrazine treatment, we designed primers (Table 3) to the genes encoding 6 (randomly selected) of 12 differentially expressed proteins and examined the transcript levels of the genes upon 0.1 µg/mL atrazine treatment from 1.5 to 12 h using qPCR. Figure 2 showed that the transcript levels of ANP32A and PDK were elevated by 1.7–3.8 fold after 1.5–3 h exposure to atrazine as compared to the control. In comparison with ANP32A transcript, a significantly delayed response was observed for HSPβ1 transcription, which was elevated by about 2 fold only after 6 h atrazine treatment. Interestingly, PKD was activated at as early as 1.5 h post treatment. In agreement with the 2D gel data, the transcript levels of NME1, TAGLN2 and PFN1 were inhibited by atrazine and the attenuation of the transcripts was a very early event. Nevertheless, the general tendency of the 6 gene transcript levels (3 up- and 3 down-regulated) was well in line with the protein levels registered in 2D gels (Table 2 and Figure 1B).

### 2.6. Verification of ANP32A Expression by Western blotting and Immunofluorescence Cell Staining

**Figure 3:**
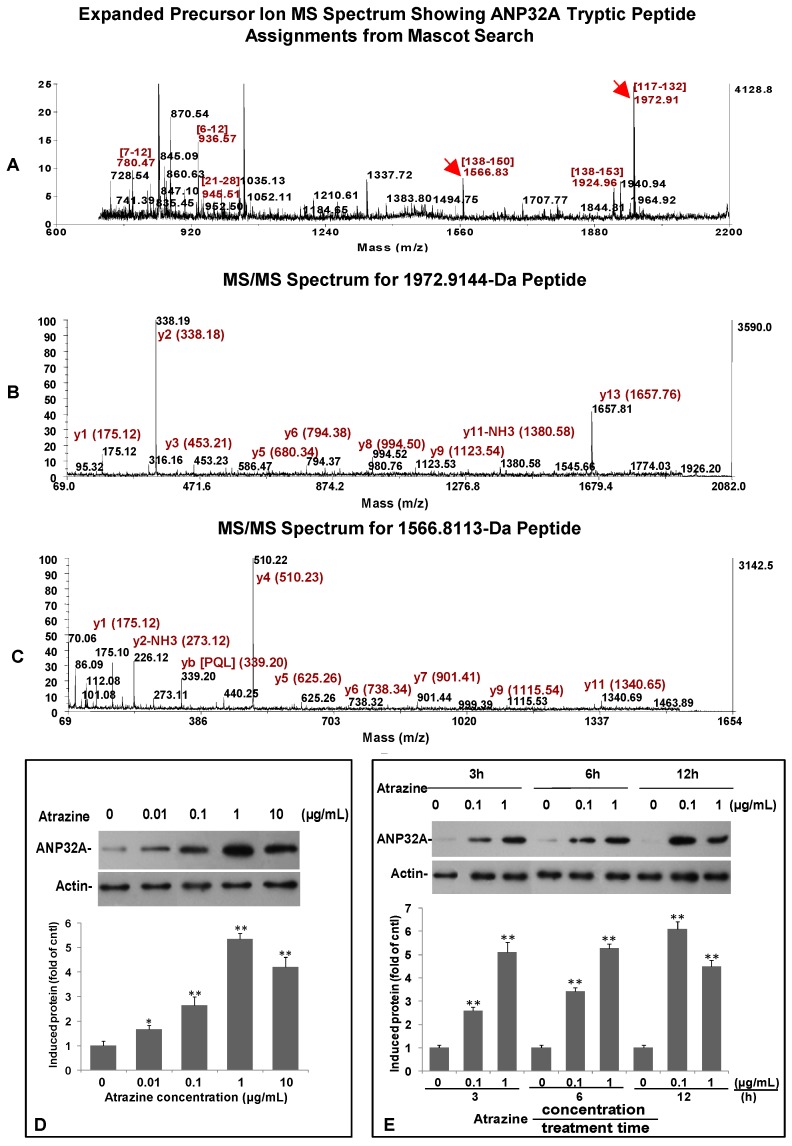
MS/MS and Western blot analysis of ANP32A in MCF-10A cells treated with or without atrazine for the indicated time periods. (**A**) Expanded precursor ion MS spectrum showing ANP32A tryptic peptide assignments from Mascot search. Two peptide fragments (1972.91 and 1566.83), marked with red arrow respectively, were subjected to further analysis; (**B**) MALDI TOF/TOF MS/MS spectrum for 1972.91 Da tryptic peptide (K.SLDLFNCEVTNLNDYR.E); (**C**) MALDI TOF/TOF MS/MS spectrum for 1566.81 Da tryptic peptide (K.LLPQLTYLDGYDR.E). Western blot analysis of dose (**D**) and time (**E**) responses of ANP32A to atrazine treatment. For the dose-response, cells were treated with indicated concentration of atrazine for 3 h. Western blot images in D and E are the representative images of blots and are subjected to densitometric analysis for the fold changes of ANP32A induced by atrazine in MCF-10A cells. The membrane was reprobed with an antibody to actin for comparison of equal protein load. Data = mean ± SD, * *p <* 0.05, ** *p <* 0.01 * versus* control.

To confirm differentially expressed proteins induced by atrazine treatment registered in 2D gel analysis and identified by MALDI-TOF/TOF MS and MS/MS analysis (Figure 1, Figure 2 and Figure 3), Western blotting and immunofluorescence staining were performed for the most modulated phosphoprotein ANP32A (Figure 3A–C). Western blot analysis revealed that in the dose response analysis, intracellular ANP32A levels started to increase at 0.01 µg/mL atrazine for 3 h and reached a maximum level (>5 fold of control) at 1.0 µg/mL atrazine (Figure 3D). The temporal expression pattern revealed that, when treated with 0.1 µg/mL, ANP32A began to increase at 3 h (approximate 2.5 fold of control) and reached the highest level (>6 fold of control) at 12 h, while, when treated with 1.0 µg/mL, ANP32A expression reached the peak level (>5 fold of control) at 6 h and then decreased at 12 h (Figure 3E). Western blot analysis showed that the response of ANP32A to atrazine treatment was dose and time dependent, a result consistent with what registered in 2D gel analysis.

**Figure 4:**
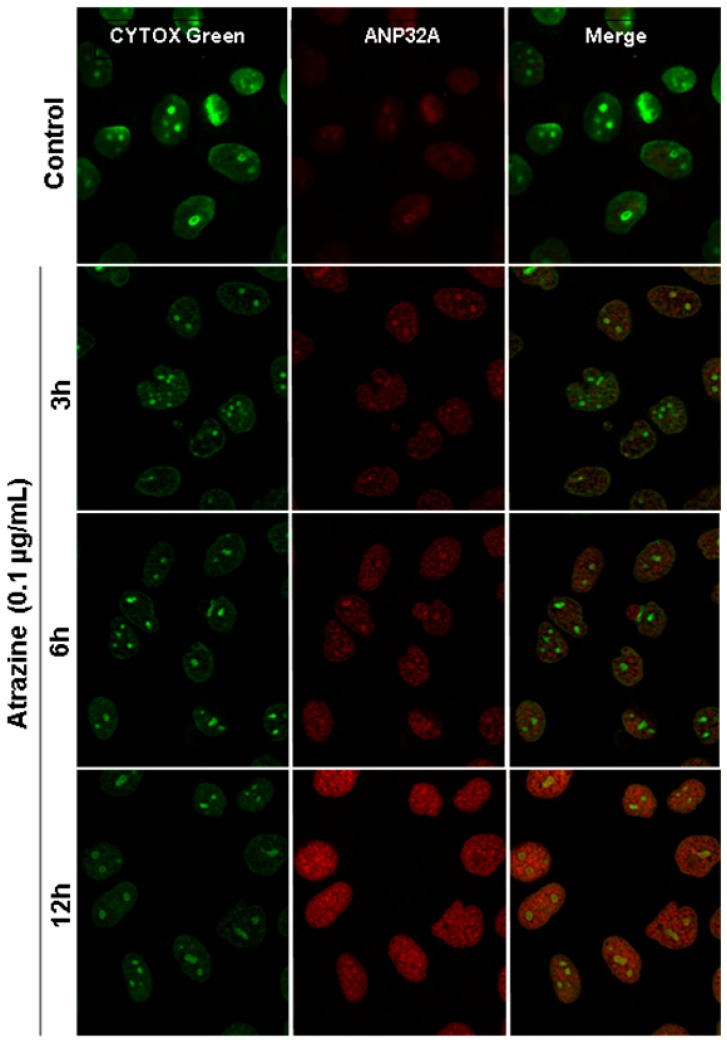
Subcellular localization of ANP32A in MCF-10A cells treated with 0.1 µg/mL atrazine for the indicated time periods. ANP32A antibody was indirectly labeled with Alexa Fluor 568 secondary antibody (red) and cells were mounted with ProLong Gold anti-fade with SYTOX Green nucleic acid stain (green). All images were captured using a Carl Zeiss confocal microscope with the same exposure time.

Although ANP32A is predominantly located in nuclei, recent reports showed that it could also relocate to cytoplasm and promote apoptosis by stimulating the apoptosome [39]. Thus, to localize the atrazine-induced overexpression of ANP32A, the immunofluorescence staining with a specific ANP32A antibody was performed. Confocal microscope images illustrated that atrazine treatment increased ANP32A expression significantly in the MCF-10A cells at a concentration as low as 0.1 µg/mL and the increased phospho-ANP32A was primarily located in the nucleus (Figure 4). The trend of the increase in immunofluorescence signal (Figure 4) was consistent with that displayed by western blot (Figure 3C,D).

NME1 and ANP32A that were differentially expressed after the atrazine treatment were located in the nucleus (Table 2). NME1 is relevant to the synthesis of nucleoside triphosphates and ANP32A is a transcriptional repressor. Both may be involved in many cellular processes, including proliferation, differentiation, and signal transduction. 2D gel analysis showed that ANP32A was one of the most modified phosphoproteins by atrazine (Figure 1A). Western blot and immunofluorescence staining analysis using specific ANP32A antibody (Figures 3D,E and Figure 4) revealed that ANP32A protein level in the atrazine-treated MCF-10A cells was elevated at the range of 0.1 to 1.0 µg/mL (Figure 3D), and declined at 10 µg/mL. The decrease of ANP32A at a relatively high atrazine level may be due to the decreased cell viability or apoptosis. ANP32A is a nuclear protein and a member of the ANP32 family of acidic, leucine-rich, nuclear phosphoproteins found in cells capable of self-renewal [40]. Recent studies indicated that apoptotic stimulation can induce translocation of ANP32A from the nucleus to the cytoplasm, and promotes apoptosis by activating apoptosome [39]. However, in our study, although atrazine provoked elevation of ANP32A shortly after exposure to atrazine, we did not observe its translocation from nucleus to cytoplasm, even when ANP32A levels were elevated to a very high level at 12 h post treatment (Figure 4). This may suggest that the induced ANP32A by atrazine in MCF-10A cells probably plays a minor role, if any, in apoptosis.

It is noticeable that exposure to atrazine evokes acetylcholinergic and dopaminergic systems dysfunction in the brain. Following fetal exposure to atrazine causes age-dependent dopaminergic dysfunction in SD rats [41]. A recent study showed that the expression of ANP32A is tightly regulated in neuronal development and directly regulates expression of the neurofilament light chain, an important neuron-specific cytoskeletal gene, by binding to the promoter of this gene and modulating histone acetylation levels [42]. ANP32A may provide the link between atrazine exposure and brain dysfunction. In contrast to its pro-apoptotic and transformation inhibition functions, ANP32A is considered as an oncogenesis protein when it is highly overexpressed in cancer cells [43]. Furthermore, ANP32A was speculated to foster increased malignancy when it is highly expressed in malignant prostatic adenocarcinomas [44]. Even though there is no clear relationship established between elevated ANP32A and tumoregenesis, it was reported that ANP32A increased in 22Rv1 and PrEC prostate cells after treatment with a well-known carcinogen benzo(a)pyrene [45]. Thus, further studies are needed to investigate the role of ANP32A in tumorgenesis of carcinogen in human breast cells.

Epidemiologic studies have linked long-term exposure of triazine herbicides to increased risk of ovarian cancer in female farm workers in Italy [26] and to breast cancer in Kentucky [27]. In addition, atrazine was also reported to lead to tumor development in the mammary gland and reproductive organs of female F344 rats [28] and to cause an earlier onset of mammary and pituitary tumors [29] in Sprague-Dawley rats, a typical response to exogenously administered estrogens [30]. Heat shock protein and the peroxiredoxin family proteins are involved in minimizing the stress-induced damage [46]. In this study, we also observed that HSPβ1, HSPA4, and PRDX1 were elevated by atrazine treatment, suggesting that the oxidative stress was induced in the treated MCF-10A cells. Oxygen is considered as a major indicator in the declining tissue associated with age, degenerative diseases [47], apoptosis and cancer [48]. The ability of atrazine or other herbicides to generate reactive oxygen species (ROS) has been previously described in goldfish [49]. The oxidative stress induced by atrazine may account for or contribute to its toxicity in human cells. Increased heat shock protein levels were observed in the MCF-10A cells exposed to atrazine. In addition to chaperone function, HSPs have been reported to be overexpressed in various cancers [50]. HSPβ1 was considered as an estrogen responsive marker in breast cancer tissues [51] and shown to be transcriptionally induced by estrogen or other steroid hormones, which was possibly due to the identity between HSPβ1 and the estrogen receptor of the related 29-kDa protein P29 [52]. Interestingly, atrazine is considered as an estrogen-like endocrine disrupting chemical (EEDC). This study indicated that the mRNA of HSPβ1 was upregulated by atrazine. Thus, it is reasonable to speculate that atrazine-induced HSPβ1 expression in MCF-10A cells may result from its estrogen-like endocrine disrupting effects.

Data also suggested that atrazine interferes with the energy metabolism in MCF-10A cells as pyruvate kinase and NME1 were modulated (Figure 1 and Table 2). Pyruvate kinase, a member of the glycolytic protein family, was up-regulated by atrazine, suggesting the higher energy demand in atrazine-treated cells. Furthermore, although pyruvate kinase is an energy regulator induced by starvation, it could also be induced by DNA damage stress or activated by ataxia telangiectasia and Rad3 related (ATR) and ataxia telangiectasia mutated (ATM) protein kinases, central checkpoint proteins in DNA damaged cells [53]. Several studies have demonstrated the ability of atrazine to induce genetic impairment in humans [54] and animals [55,56] resulting in cell damage or apoptosis [57].

## 3. Experimental Section

### 3.1. Atrazine Preparation

A stock solution at a concentration of 10 mg/mL was prepared by dissolving atrazine (98% purity, Superlco, Bellefonte, PA, USA) in 100% DMSO. Before treatment, the stock solution was diluted to 1.0, 0.1, and 0.01 mg/mL with DMSO, and then added to the cell culture medium (dilution 1/1000) to obtain the final concentrations of 10, 1.0, 0.1 and 0.01 μg/mL, respectively.

### 3.2. Cell Culture

MCF-10A cells (purchased from ATCC) were grown as a monolayer in TC175 flasks at 37 °C under a humidified atmosphere of 5% CO_2_ in an incubator and maintained in MEGM (Lonza, Walkersville, MD, USA) containing 10 ng/mL hEGF, 5 μg/mL insulin, 0.5 μg/mL hydrocortisone gentamicin and amphotericin-B. Before MEGM was used, bovine pit extract (BPE) (13 mg/mL, Lonza, Walkersville, MD, USA) was added into MEGM (a final concentration of 0.4% BPE). For atrazine treatment, the cells (1.0 × 10^6^) were seeded in 100 × 15 mm sterile petri dishes (Fisher Scientific, St Louis, USA) for 24 h and then exposed to either 0.1% DMSO (control) or the indicated concentrations of atrazine.

### 3.3. Identification of Differentially Expressed Proteins

MCF-10A cells of three dishes were harvested and washed with PBS twice for each treatment. After removing the supernatant, the pellet was re-suspended in 150 μL 10 mM Tris-HCl (pH 7.0), sonicated on ice for 45 s, and centrifuged at 16,000× *g* for 10 min at 4 °C. The supernatant (150 μL) was transferred into 1.5 mL tubes and added with 600 μL of methanol, 150 μL of chloroform, and 450 μL of ultrapure water for delipidation and subsequent desalting processes following the manufacturer’s protocol of Pro-Q^®^ Diamond phosphoprotein gel stain (termed Pro-Q stain thereafter) (Molecular Probes, Invitrogen, Catalog No. MP33300). After mixed by a vortex, the samples were centrifuged at 16,000× *g* for 5 min at 4 °C, and the upper liquid was discarded. The white precipitation disc that formed between the upper and lower phases was then added with 450 μL of methanol, mixed by vortex, and centrifuged at 16,000× *g* for 5 min at 4 °C. The supernatant was discarded, and the pellets were air-dried for 10 min.

The dried pellets were re-dissolved in a rehydration buffer [7 M urea, 2 M thiourea, 4% (w/v) CHAPS, 40 mM DTT, 0.5% (v/v) IPG buffer 3-10 NL (GE healthcare), 0.001% (w/v) bromphenol blue]. Protein concentration was determined using a Bio-Rad protein bioassay kit (reagent A: catalog No. 500-0113; reagent B: catalog No. 500-0114). The IPG strip (11 cm, pH 3–10 NL from Bio-Rad) was rehydrated with 350 μL rehydration buffer containing 600 mg total protein. Isoelectric focusing (IEF) was carried out at 20 °C in an IEF Protean Cell (Bio-Rad) as the following as: 250 V for 1 h, 500 V for 1 h, 1000 V for 2 h, linear to 8000 V for 1 h, and 8000 V for 5 h. The strips were then soaked for 15 min in an equilibration buffer [6 M urea, 30% (v/v) glycerol, 2% SDS, 50 mM Tris–HCl (pH 8.8), and 1% DTT] and for an additional 15 min in the same buffer with 2.5% iodoacetamide instead of DTT. The second dimension was run on 12% SDS polyacrylamide gels.

The gels were stained with Pro-Q stain following the manufacture’s instruction. In brief, the gels were fixed in 50% ethanol and 10% acetic acid overnight, washed three times with deionized water for 15 min, incubated in Pro-Q Diamond for 120 min in dark, and then destained with three washes in 50 mM sodium acetate (pH 4.0) containing 20% acetonitrile. After two washes with deionized water, the phosphoprotein images were acquired with a Typhoon Variable Model Images (FLA-5000, FUJI FILM) with a 532 nm excitation and 580 nm band pass emission filter, and the gels were then stained with CB (G-250) for protein spot detection.

### 3.4. In-Gel Protein Digestion

Both Pro-Q and CB-stained 2D gels images between the atrazine-treated and control samples were compared, and differentially displayed protein spots were identified. By overlapping images of Pro-Q stain with CB stain, the differentially expressed phosphoproteins and total protein spots on the CB stained gels were localized and excised using a 1 mm diameter metal punch. The excised gel pieces were subjected to in-gel digestion following the standard protocol provided by the MU Proteomics Center as described previously [58]. In brief, the excised gel pieces were placed in a clean 1.5 mL Safe-Lock Eppendorf tubes (catalog No. 022363204) and destained three times with 500 µL of acetonitrile/100 mM ammonium bicarbonate solution [50/50 (v/v)] for 15 min with agitation and washed briefly with 500 µL of acetonitrile. The gel pieces were then dehydrated for 20 min with 500 µL of acetonitrile at room temperature under agitation. The dehydrated gel plugs were subsequently rehydrated for 2 h at 4 °C in 5 μL of modified L-1-Tosylamide-2-phenylethyl chloromethyl ketone (TPCK) treated porcine trypsin solution (20 µg/mL) (Trypsin Gold, mass spectrometry grade, 17,000 U/mg, catalog No. V5280, Promega, Madison, WI, USA) in 40 mM ammonium bicarbonate/10% acetonitrile. Subsequently, the trypsin solution was replaced with 15 μL of 40 mM ammonium bicarbonate/10% acetonitrile, and the proteins were digested overnight at 37 °C. The digests were acidified by adding 4 μL of the extraction solvent and transferred into 500 μL tubes. Each gel piece was extracted twice with 10 μL of acetonitrile/water/10% trifluroacetic acid solution [600/300/100 (v/v/v)] for 10 min with gentle agitation. Extracts from the same sample were pooled, snap frozen in liquid N_2_, and stored at −80 °C.

### 3.5. Trypsin Digest Processing and Sample Target Preparation

The samples were removed from −80 °C and dried on a lyophilizer. After the first drying, the samples were each dissolved in 10 µL water, refrozen, and dried a second time by lyophilization.

For MALDI TOF/TOF MS analysis, the dried samples were dissolved in “reconstitution solvent” consisting of 700/290/10, *v*/*v*/*v*, acetonitrile/water/88% formic acid. Samples 4 and 25 through 53 were dissolved in 2 µL reconstitution solvent. All of the other samples were initially dissolved in 1 µL of reconstitution solvent. An equal volume of alpha-cyano-4-hydroxycinnamic acid (CHCA) matrix solution was then added to the sample solution. Aliquots of the mixture (0.4 µL each) were deposited on a polished stainless steel target (ABI01-192-6-AB). Crystallization of the mixture proceeded under ambient conditions. The crystals were not washed. CHCA solution was prepared at a concentration of 5 mg/mL in 500/455/20/25 (*v*/*v*/*v*/*v*) acetonitrile/water/10% trifluoroacetic acid (aq)/0.4 M (aq) ammonium dihydrogen phosphate.

### 3.6. MALDI TOF/TOF MS and MS/MS Analysis

Spectra were acquired on an Applied Biosystems Inc. (now AB Sciex) 4700 MALDI TOF/TOF mass spectrometer with a 355 nm Nd:YAG laser (200-Hz) in the positive ion delayed-extraction reflector MS or positive ion MS/MS mode. The MS spectra were acquired over the mass range 700–4500 Da (sum/average of 100 20-shot sub-spectra, the first 2 shots rejected from each sub-spectrum). An accelerating voltage of 20 kV combined with a 70% extraction grid voltage and an extraction delay time set for a focus mass at 2000 Da, resulted in a mass resolving power (FWHM) ranging from 9000 to 16,000 across the mass range of the analysis. The external calibration was based on spectra for the Sequazyme peptide standards mix (Applied Biosystems, Inc., Grand Island, NY, USA) spotted on 6 positions surrounding the sample spots on the target. Automatic internal calibration was performed on the MS spectra post-acquisition if at least 2 of the following trypsin autolysis peptides were present in the spectrum at sufficient intensity (S/N 20): [M+H]^+^ 842.5100 Da, 1045.564, and 2211.105 Da. The external default calibration was applied to those spectra which did not have adequate signal from the trypsin autolysis peptides. Spectra were processed with Applied Biosystems’ 4000 Series Explorer software (version 3.6).

The 1-KV peptide unimolecular decomposition spectra, or MS/MS, were obtained with or without CID gas as indicated and with metastable ion suppression. Resolving power for ions in the MS/MS spectra ranged from 1500–5000 across the mass range from 60 Da to the mass of the precursor ion. The mass gate for precursor ion selection was set to allow into the collision cell, ions from 2.0 Da below to 2.5 Da above the precursor ion mass. External mass calibration for the MS/MS was based on fragment ion masses from the peptide ACTH [18–39] ([M+H]^+^ 2465.1990 Da). Note that commonly observed contaminant ions and trypsin autolysis peptides were automatically excluded from MS/MS analysis (568.14, 832.3, 842.51, 856.52, 870.541, 1045.564, 1794.8, 1826.8, 1940.935, 2211.105, 2225.12, 2239.136, 2283.181, 2284.19, 2299.18, 2300.18, 2807.315 and 3337.758 Da). Peptides that were potentially oxidized or sodium- or potassium-adducted forms of other peptides present in the spectrum were also excluded from MS/MS analysis. Additional masses of common keratin tryptic peptides were added to the MS/MS exclusion lists over the course of analysis of the samples.

### 3.7. MALDI TOF/TOF MS Data Analysis

Peak lists from the combined “MS and MS/MS” spectra were submitted via the Applied Biosystems’ GPS Explorer software (Vers. 3.6) to database searches with Matrix Sciences’ Mascot program (version 2.3 residing on a local server). The data were queried against the NCBInr Human protein database (last updated on or after 6 September 2010). Parameters limited the initial search to trypsin digestion with up to one missed cleavage allowed and required cysteine modification with iodoacetamide (Ccarbamidomethyl = Ccarboxamidomethyl = Ccam). Pyroglutamate formation at peptide N-terminal glutamines and glutamic acids was allowed as a variable modification as was methionine oxidation. The molecular weight of the source protein was not limited. The mass error tolerance was set to ±25 ppm for peptide masses in the MS spectrum and ±0.1 Da for fragment ions in the MS/MS spectra. A maximum of 500 ions with S/N ≥ 20 was submitted from the MS for the searches. MS/MS searches accepted up to 250 fragment ions with S/N ≥ 10. Masses (±100 ppm) of the major trypsin autolysis peptides and common background contaminants were excluded from the peak lists submitted for the searches (832.3, 842.51, 870.54, 1045.5642, 1826.6, 2211.1046, 2239.1359, 2299.1756, 2807.31, 3337.758 Da).

### 3.8. Quantitative Real-Time PCR (qPCR) Characterization

The cells were incubated with or without 0.1 µg/mL atrazine for 1.5, 3, 6, 12 h, respectively, and total RNA was isolated using Trizol reagent (Invitrogen) according to the manufacturer's instructions. RNA concentration was determined by measuring the absorbance at 260 nm with a spectrophotometer (NanoDrop 2000, Thermo Scientific, Wilmington, USA). About 2 μg of total RNA per sample was used for the first-strand cDNA synthesis. The synthesis was primed using oligo (dT) based on the SuperScript First-Strand synthesis kit (Invitrogen, catalog No. 11904-018). The synthesized cDNAs were used as templates for qPCR estimation of gene transcription in MCF-10A cells. The atrazine-responsive genes and the corresponding primers used in qPCR are listed in Table 1. qPCR amplification and analysis were carried out using an iQTM5 Real-Time PCR detection system (BIO-RAD). The final volume of reaction was 20 µL using iQTM SYBR Green Supermix (BIO-RAD). The qPCR reaction was held at 95 °C for 10 min followed by 45 cycles at 95 °C for 15 s, then 62 °C for 1 min. The specificity of the SYBR Green PCR signal was confirmed by melting curve analysis and agarose gel electrophoresis. The mRNA expression was quantified using the comparative CT (the qPCR cycle number that crosses the signal threshold) method [59]. The CT of the housekeeping gene GAPDH was subtracted from CT of the target gene to obtain ΔCT. The normalized fold changes of the target gene mRNA expression were expressed as 2-ΔΔCT, where ΔΔCT is equal to ΔCT treated sample-ΔCT control.

### 3.9. Western Blot Verification

MCF-10A cells were treated with each of three concentrations of atrazine for 3, 6 and 12 h, harvested, and lysed in 40 µL lysis buffer (8 M Urea, 50 mM Tris, pH 7.5, 150 mM 2-Mercaptoethanol). With a 1:10 dilution of the samples, protein concentrations were determined as described above. Protein (15 µg each sample) was separated on 12% SDS-polyacrylamide gels, transferred to PVDF membranes (0.4 μm, Millipore), immunoblotted with anti-ANP32A (C-terminal) (ABGENT, catalog# AP9520b) at a dilution of 1:2000, and then visualized by enhanced chemiluminescence (Super signal West Pico, Thermo Scientific, Rockford, IL,USA).

### 3.10. Immunofluorescence Staining

MCF-10A cells were grown on cover slips (pre-coated with 40 µg/mL poly-L-lysine) kept in 35 mm petri dishes and incubated with/without 0.1 µg/mL atrazine for 3, 6, and 12 h respectively. The cell layers were washed with 2 mL PBS 3 times and fixed for 20 min in 4% paraformaldehyde at room temperature, followed by 2 washes with PBS, 5 min each. The cells were then permeabilized for 3 min with 0.25% Triton X-100 in PBS, washed twice in 2 mL PBS, and blocked for 1 h with 5% BSA in PBS. The cells were incubated with anti-ANP32A (C-terminal) (ABGENT, catalog# AP9520b) primary antibodies (diluted 1:200 with 1% BSA in PBS) at 4 °C overnight, washed three times with PBS, and incubated with secondary antibodies (goat anti-mouse-Alexa Fluor 568) at a 1:400 dilution for 2 h at room temperature. After 3 washes with PBS, 5 min each, cover slides were incubated with SYTOX Green nucleic acid stain (Molecular Probes, catalog No. S7020) at a 1:1000 dilution for 10 min, followed by three washes with PBS before being mounted on cover slides with Aqueous Mounting Medium (Permafluor). Images were taken using a Carl Zeiss confocal microscope at the Molecular Cytology Core Facility at University of Missouri-Columbia.

### 3.11. Statistical Analysis

All data are presented as the mean ± SD of at least three independent experiments. Differences between groups were assessed by the one-way analysis of variance (ANOVA). If the variances between groups were homogenous, groups were subjected to the multiple comparison Dunnett’s test. * *p* < 0.05, ** *p* < 0.01 compared with untreated controls.

## 4. Conclusions

This study illustrated that atrazine at the environmental relevant concentrations induces differential expression of several phosphoproteins and total proteins located in various cell compartments, which are involved in many cell processes such as stress response, cell shape, gene expression regulation, and carcinogenesis. The molecular mechanism for the differential protein expression remains unclear. Applications of advanced technologies such as DNA array and proteome–secretome analysis are needed to further exploit the specific metabolic function for the differentially expressed proteins associated with atrazine treatment.
